# Prevalence and clinical association of sarcopenia among Thai patients with systemic sclerosis

**DOI:** 10.1038/s41598-022-21914-w

**Published:** 2022-10-28

**Authors:** Arthitaya Sangaroon, Chingching Foocharoen, Daris Theerakulpisut, Kannikar Srichompoo, Ajanee Mahakkanukrauh, Siraphop Suwannaroj, Patpiya Seerasaporn, Chatlert Pongchaiyakul

**Affiliations:** 1 Department of Medicine, Faculty of Medicine, Khon Kaen University, Khon Kaen, Thailand; 2 Department of Nuclear Medicine, Faculty of Medicine, Khon Kaen University, Khon Kaen, Thailand; 3 Department of Rehabilitation Medicines, Faculty of Medicine, Khon Kaen University, Khon Kaen, Thailand; 4 Division of Endocrinology and Metabolism, Department of Medicine, Faculty of Medicine, Khon Kaen University, Khon Kaen, Thailand

**Keywords:** Medical research, Rheumatology

## Abstract

Patients with systemic sclerosis (SSc) have some potential factors associated with an increased risk of sarcopenia. There has been currently no study to examine such associations in SSc patients. We aimed to determine the prevalence of sarcopenia among SSc patients and examine its association with clinical manifestations and laboratory tests. A cross-sectional study was conducted in 180 adult SSc patients at the Scleroderma Clinic, Khon Kaen University, Thailand, between July 2019 and April 2020. Clinical data, laboratory tests for inflammatory markers, serology, hormone, body composition by dual-energy X-ray absorptiometry, handgrip strength, functional lower extremity strength, and usual gait speed were collected and measured. Sarcopenia was defined according to the criteria of the Asian Working Group for Sarcopenia. One hundred and eighty patients were recruited. Ninety-four cases (52.2%) were the diffuse cutaneous SSc subset. The respective mean age and duration of disease was 58.8 ± 9.4 and 6.2 ± 5.3 years. Sarcopenia was revealed in 41 SSc patients for a prevalence of 22.8% (95% CI 12.1–34.8), while the prevalence was higher in patients with the diffuse cutaneous SSc (dcSSc) compared to the limited cutaneous SSc. BMI at the onset of SSc and C-reactive protein > 5 mg/dL were significantly associated with sarcopenia with a respective OR of 0.60 (95% CI 0.48–0.75) and 3.18 (1.06–9.54). Sarcopenia is common in patients with SSc, but the prevalence is more pronounced in the dcSSc. Inflammatory markers, particularly the CRP level, are strongly associated. BMI at the onset had a negative association with sarcopenia among SSc patients.

## Introduction

Systemic sclerosis (SSc) is a rare systemic connective tissue disease characterized by skin thickness. Fibrosis is a classical pathological finding that reveals in both skin and internal organ^1^. The diffuse cutaneous SSc (dcSSc) subset is significantly associated with bad prognosis^2,3^. The patient with SSc is likely to be at risk for developing sarcopenia. Previous studies demonstrated that several factors for sarcopenia—including malnutrition related to gastrointestinal involvement, chronic inflammation, drugs such as steroid therapy, physical inactivity, and terminal organ failure—also present in patients with SSc^4,5^.

Sarcopenia is an age-related disease described as a progressive loss of muscle mass and function. A recent study—using definitions from The European Working Group on Sarcopenia in Older People (EWGSOP) and the Asian Working Group of Sarcopenia (AWGS)—reported the prevalence of sarcopenia in healthy adults aged ≥ 60 years was 10% and that rates are comparable between men and women^6^. The prevalence of sarcopenia varied based on ethnicity, measurement methods, and study populations. For instance, Shafiee et al.^6^ reported that the prevalence of sarcopenia was higher among non-Asian patients than Asian individuals in both sexes and higher when using the Bio-electrical impedance analysis (BIA) to measure muscle mass compared with dual-energy X-ray absorptiometry (DXA) (a respective 19% vs. 10% in men, and 20% vs. 11% in women)^6^. Another study demonstrated that the prevalence of sarcopenia was highest in nursing-home individuals (51% and 31% in men and women, respectively) compared with community-dwelling individuals (11% and 9% in men and women, respectively), and hospitalized individuals (23% and 24% in men and women, respectively)^7,8^.

Siegert et al.^4^ reported that, when using the EWGSOP criteria, the prevalence of sarcopenia in German patients with SSc was 22.5%, and this rate increased with age. The authors found that the potential predictors of sarcopenia—such as disease duration, numbers of medications, numbers of comorbidities—were not different between SSc patients with sarcopenia and non-sarcopenia^4^. In 62 Italians with SSc, Corallo et al.^9^ found a prevalence of sarcopenia of 42% by Relative Skeletal Mass Index and 55% by handgrip strength. The clinical predictors of sarcopenia were elderly individuals, long disease duration, malnutrition status, extensive skin thickness, and esophageal involvement^9^. Other studies^7,10^, however, suggested that other factors—longer disease duration, abnormal body compositions (low body mass index—BMI) and lean mass (LM), and relative skeletal muscle mass index—SMI), lung and skin involvements—were associated with sarcopenia in patients with SSc.

To date, there is a paucity of evidence on the association between sarcopenia and relevant parameters in patients with SSc, including clinical features of SSc, serology, inflammatory markers, and concomitant medications. The current study aimed to determine the prevalence of sarcopenia and examine the associations between its clinical factors and sarcopenia in adult Thai patients with SSc.

## Methods

### Setting and subjects

The study was designed as a cross-sectional investigation among adult patients with SSc who, between July 2019 and April 2020, attended the Scleroderma Clinic, Khon Kaen University, Khon Kaen, Thailand. Excluded from the study were patients with (1) overlap syndrome; (2) neurological diseases (including neurodegenerative, motor neuron diseases, cerebrovascular disease, multiple sclerosis, muscular dystrophy, spinal cord disease, anterior horn cell disease, or peripheral neuropathy); (3) immobilization syndrome or unable to walk due to severe joint contracture or painful ulcer affecting the measurement of muscle strength; (4) active myositis (muscle weakness with rising of muscle enzyme, inflammatory myositis from muscle biopsy, and abnormal electromyography); (5) severe infection or sepsis; (6) any stage of cancer; or, (7) pregnant or lactating.

### Measurements

The participants were invited to meet a research nurse who administered the questionnaire and informed consent form. Demographic data were recorded, including age, sex, underlying diseases, smoking and alcohol intake patterns, and current medications. Clinical assessments were performed in all eligible patients, including symptoms on the visit such as Raynaud’s phenomenon, digital ulcers, skin tightness, synovitis, hand deformity, tendon friction rub, stomach and esophageal involvement.

Blood samples were collected, and measurements included serology (anti-centromere antibody (ACA), anti-topoisomerase I antibody (ATA)), complete blood count, C-reactive protein (CRP), erythrocyte sedimentation rate (ESR), vitamin D [25,(OH)D] level, testosterone (in men), estradiol (in women), cortisol, and thyroid function test (free T4, free T3, and thyroid-stimulating hormone—TSH). Upon enrollment, the following were measured body composition using dual-energy X-ray absorptiometry (DXA), handgrip strength, functional lower extremity strength, and usual gait speed.

### Body composition

Body composition (muscle mass and fat mass) for each patient was measured by DXA (Lunar Prodigy bone densitometer, GE Healthcare, Madison, WI, USA; CV = 1% for whole body values). According to the DXA results, we calculated the appendicular skeletal muscle mass (ASM) for each patient as the sum of the upper and lower limb muscle mass without bone and fat tissue. Appendicular skeletal mass index (ASMI) was calculated as the ASM divided by height squared (ASM/height^2^, kg/m^2^)^11,12^. According to the Asian Working Group for Sarcopenia (AWGS), the cut-off point for ASMI for low muscle mass is < 7.0 kg/m^2^ for men and < 5.4 kg/m^2^ for women^11,13^. The fat free mass index (FFMI) was calculated to identify muscle loss using the fat free mass divided by height squared. The cut-off value for low FFMI is < 15.5 kg/m^2^ for men and < 12.6 kg/m^2^ for women^14^.

### Muscle strength assessment

#### Handgrip strength test

Handgrip strength (in kg) was measured using the Takei T.K.K.5001 handgrip dynamometer (Takei Scientific Instruments Co., Ltd, Tokyo, Japan). Participants stood in a neutral position, arms by their side with full elbow extension, then the participants were asked to press the dynamometer for 5 s for each measurement. All measurements were carried out alternatively between the left and right hands three times with no rest. Maximum strength values for both hands were recorded. According to the AWGS 2019, the cut-off point for low handgrip strength is < 28 kg for men and < 18 kg for women^15^.

### Physical performance assessment

In the study, a 6-m gait speed was used for the assessment of physical performance. Each participant walked normally with and without instruments, depending on the safety and comfort of the participant^16^. The test was repeated thrice, and each recorded time calculated and used for interpretation. The cut-off point for low gait speed was 1.0 m/s for both men and women^15^.

Both muscle strength and physical performance assessments were conducted by experienced physical therapists, nurses, and research assistants. Each test was performed only by the participant when he/she felt comfortable and had normal vital signs. A participant was removed from the study if any adverse events resulted from the tests (i.e., severe muscle injury, dyspnea uncorrected after rest or requiring oxygen therapy), or they could not complete all the measurements.

### Operational definitions

SSc was diagnosed according to the 2013 ACR/EULAR Classification for Scleroderma^17^ and classified as per LeRoy et al.^18^ as dcSSc or limited cutaneous SSc (lcSSc). Sarcopenia was defined according to the criteria of the Asian Working Group for Sarcopenia (AWGS) 2019^15^. Participants who met the criteria for both low handgrip strength or low gait speed and low muscle mass were considered to have “sarcopenia”, and participants with low handgrip strength, low gait speed, and low muscle mass as having “severe sarcopenia”.

Pulmonary involvement was fulfilled when interstitial fibrosis is detected by high resolution computed tomography (HRCT). In this study, pulmonary arterial hypertension (PAH) was diagnosed by: (a) the mean pulmonary arterial pressure (mPAP) is > 20 mmHg at rest; and, (b) a pulmonary artery wedge pressure of ≤ 15 mmHg and a pulmonary vascular resistance of ≥ 3 Wood units, as confirmed by right heart catheterization^19^. According to the WHO definition, low body mass index (BMI) was < 18.5 kg/m^2^ in the present study^20^. Anemia was defined as < 12 g/dL in non-pregnant women and < 13 g/dL in men^21^. High CRP and ESR were considered when the levels were > 5 mg/dL and > 25 mm/h, respectively^22,23^. Serum 25(OH)D level < 30 ng/mL was defined as vitamin D insufficiency^24,25^. The definition of a low cortisol level was fulfilled when it was < 5 µg/dL. The definition of high thyroid-stimulating hormone (TSH) was fulfilled when it was > 4.2 mIU/L. The definition of hypothyroidism was fulfilled when TSH was > 4.2 mIU/L, and Free T4 was < 0.93 ng/dL.

### Sample size calculation

The sample size was initially determined based on the prevalence of sarcopenia and the study’s primary objective. Based on an estimated 22%, the prevalence of sarcopenia in SSc^4^ (with a precision of 10% and a confidence level of 95%) required at least 66 cases be included. The secondary objective of the study sought to determine the factors associated with sarcopenia in SSc. It referred to a ratio of 1 suspected associated factor using an equation. According to the literature review, ten sarcopenic patients would be needed to study at least four factors associated with sarcopenia^10,26^. Thus, at least 180 SSc patients were required for the current study.

### Statistical analysis

Continuous variables were presented as means and standard deviations. The prevalence of sarcopenia was expressed as a percentage and 95% confidence interval (95% CI). We compared SSc patients with sarcopenia to those without sarcopenia. A Chi-square test or Fischer’s exact test was performed to evaluate the association between dichotomous variables. A Student’s t-test or Wilcoxon-Mann–Whitney test was used to examine differences for continuous data between two groups as appropriate. The association between sarcopenia (dependent variable) and the independent variables (SSc characteristics and laboratory tests) was expressed as the odds ratio (OR) and its corresponding 95% CI. A multivariate logistic regression model was run, and the following covariables were included based on their clinical relevance. Any additional significant variables (*p*-value < 0.1) from the univariate analysis were also included in the model. Statistical significance was defined as a *P*-value < 0.05. Statistical analyses were performed using STATA version 16.0 (StataCorp., College Station, TX, USA).

### Ethics approval and consent to participate

The Human Research Ethics Committee of Khon Kaen University reviewed and approved the study as per the Helsinki Declaration and the Good Clinical Practice Guidelines (HE621103). All eligible patients signed informed consent before enrollment.

## Results

One hundred and eighty patients were recruited in the study, for which the female to male ratio was 1.9:1. The majority of patients (94 cases; 52.2%) had the diffuse cutaneous SSc subset. The respective mean age and mean duration of disease was 58.8 ± 9.4 and 6.2 ± 5.3 years. Sarcopenia—as defined by the AWGS definition—was revealed in 41 SSc patients for a prevalence of 22.8% (95% CI 12.1–34.8), and 73.2% (30/41) were classified as having severe sarcopenia. Around half of the patients who had sarcopenia were ≥ 60 years of age. The demographic data are presented in Table 1.

**Table 1:** Demographic data of study population.

Factor	Overall N = 180 (%)
Age at onset (years); mean ± SD	52.55 ± 10.68
Age at enrollment (years); mean ± SD	58.77 ± 9.38
**Age at enrollment**	
< 40 years	5 (2.8)
40–59 years	93 (51.7)
≥ 60 years	82 (45.6)
Duration of disease (years); mean ± SD	6.23 ± 5.27
dcSSc subset	94 (52.2%)
Male sex	61 (33.9%)
**Serology**	
ATA positive	136 (81.4%)
ACA positive	13 (7.8%)
**Comorbidities**	
Diabetes mellitus	17 (9.4%)
Hypertension	34 (18.9%)
Dyslipidemia	39 (21.7%)
**Smoking habit**	
Ex-smoking	9 (5.0%)
Current smoking	4 (2.2%)
Alcohol intake	4 (2.2%)
**Clinical at onset**	
Low body mass index (< 18.5 kg/m^2^)	26 (14.4%)
Raynaud’s phenomenon	147 (81.7%)
Digital ulcer	29 (16.1%)
Telangiectasia	20 (11.1%)
Calcinosis cutis	2 (1.1%)
Salt and peppers skin	98 (54.4%)
Edematous skin	63 (35.0%)
Tendon friction rub	21 (11.7%)
Hand deformity	15 (8.3%)
Synovitis	46 (25.6%)
Esophageal involvement	59 (32.8%)
Stomach involvement	43 (23.9%)
Pulmonary fibrosis	79 (43.9%)
Pulmonary arterial hypertension	11 (6.1%)
Renal crisis	0 (0.0%)
**Investigation at onset**	
mRSS (points); mean ± SD	9.37 ± 8.21
Anemia	81 (45.3%)
Serum albumin (g/dL); mean ± SD	4.04 ± 0.5
FVC (% predicted); mean ± SD	70.63 ± 14.20
LVEF < 50%	2 (1.4%)
**Medication at onset**	
Prednisolone	
None	66 (36.7%)
≤ 15 mg/day	107 (59.4%)
> 15 mg/day	7 (3.9%)
**Immunosuppressive drugs at onset**	
Oral cyclophosphamide	52 (28.9%)
Methotrexate	15 (8.3%)
Azathioprine	1 (0.6%)
Mycophenolate mofetil	5 (2.8%)
**Clinical at enrollment**	
Low body mass index	45 (25.0%)
Raynaud’s phenomenon	99 (55.0%)
Digital ulcer	19 (10.6%)
Telangiectasia	26 (14.4%)
Calcinosis cutis	4 (2.2%)
Salt and peppers skin	49 (27.2%)
Edematous skin	15 (8.3%)
Tendon friction rub	6 (3.3%)
Hand deformity	27 (15.0%)
Synovitis	3 (1.7%)
Esophageal involvement	44 (24.4%)
Stomach involvement	38 (21.1%)
Pulmonary fibrosis	90 (50.0%)
Pulmonary arterial hypertension	20 (11.1%)
Renal crisis	1 (0.6%)
**Investigation at enrollment**	
mRSS (points); median (IQR)	2 (0–5)
Anemia	82 (45.6%)
FVC (% predicted); mean ± SD	68.66 ± 15.03
LVEF < 50%	3 (3.2%)
High ESR	118 (65.6%)
High CRP	54 (30.3%)
Serum albumin (g/dL); median (IQR)	4.3 (4.1–4.6)
Testosterone level (mIU/L) mean ± SD	5.06 ± 1.71
Estradiol level (ug/dl); mean ± SD	17.98 ± 32.54
Vitamin D insufficiency	78 (43.3%)
Low Cortisol	40 (22.2%)
High TSH	21 (11.7%)
**Body composition**	
Lean mass (kg)	36.39 ± 7.56
Fat free mass index (kg/m^2^)	6.60 ± 3.26
Low fat free mass index	36 (20.0%)
**Medication at enrollment**	
Prednisolone	
None	94 (52.2%)
≤ 15 mg/day	86 (47.8%)
> 15 mg/day	0 (0.0%)
**Immunosuppressive drugs at enrollment**	
Oral cyclophosphamide	22 (12.2%)
Methotrexate	8 (4.4%)
Azathioprine	2 (1.1%)
Mycophenolate mofetil	33 (18.3%)

*95%CI* 95% confidence interval, *NA* data not available due to statistical limitation, *dcSSc* diffuse cutaneous systemic sclerosis, *ATA* anti-topoisomerase I antibody, *ACA* anti-centromere antibody, *mRSS* modified Rodnan skin score, *FVC* forced vital capacity, *LVEF* left ventricular ejection fraction, *IQR* interquartile range, *ESR* erythrocyte sedimentation rate, *CRP* C-reactive protein, *TSH* thyroid-stimulating hormone.

Significantly more SSc patients with the dcSSc subset that were male and positive for the ATA test had sarcopenia. The clinical manifestations at onset that were more prevalent among patients with sarcopenia than without included digital ulcer, hand deformity, low BMI (< 18.5 kg/m^2^), high mRSS, low serum albumin, and anemia. The only clinical manifestation at enrollment prevalent among sarcopenic SSc patients was high TSH. The serum albumin, lean mass, fat free mass, and fat free mass indices were significantly lower in patients with sarcopenia than non-sarcopenic patients (Table 2).

**Table 2:** Comparative variables between SSc patients with and without sarcopenia.

Factor	Without sarcopenia N = 139 (%)	With Sarcopenia N = 41 (%)	P-value
Age at onset (years); mean ± SD	52.19 ± 10.38	53.73 ± 11.69	0.42
Age at enrollment (years); mean ± SD	58.31 ± 9.29	60.36 ± 9.63	0.22
**Age at enrollment**			
< 40 years	4 (2.9)	1 (2.4)	–
40–59 years	75 (54.0)	18 (43.9)	0.97
≥ 60 years	60 (43.2)	22 (53.7)	0.74
Duration of disease (years); mean ± SD	6.11 ± 5.31	6.64 ± 5.17	0.57
dcSSc subset	64 (46.0%)	30 (73.2%)	< 0.001*
Male sex	40 (28.8%)	21 (51.2%)	< 0.001*
**Serology**			
ATA positive	102 (77.9%)	34 (94.4%)	0.02**
ACA positive	11 (8.4%)	2 (5.6%)	0.57
**Comorbidities**			
Diabetes mellitus	13 (9.4%)	4 (9.8%)	0.94
Hypertension	30 (21.6%)	4 (9.8%)	0.09
Dyslipidemia	31 (22.3%)	8 (19.5%)	0.70
**Smoking habit**			
Ex-smoking	8 (5.8%)	1 (2.4%)	0.39
Current smoking	4 (2.9%)	0 (0.0%)	0.27
Alcohol intake	4 (2.9%)	0 (0.0%)	0.27
**Clinical at onset**			
Low body mass index (< 18.5 kg/m^2^)	9 (6.5%)	17 (41.5%)	< 0.001*
Raynaud’s phenomenon	113 (81.3%)	34 (82.9%)	0.81
Digital ulcer	17 (12.2%)	12 (29.3%)	< 0.001*
Telangiectasia	16 (11.5%)	4 (9.8%)	0.75
Calcinosis cutis	2 (1.4%)	0 (0.0%)	0.44
Salt and peppers skin	69 (49.6%)	29 (70.7%)	0.01*
Edematous skin	52 (37.4%)	11(26.8%)	0.21
Tendon friction rub	13 (9.4%)	8 (19.5%)	0.08
Hand deformity	6 (4.3%)	9 (21.9%)	< 0.001*
Synovitis	34 (24.5%)	12 (29.3%)	0.54
Esophageal involvement	43 (30.9%)	16 (39.0%)	0.33
Stomach involvement	37 (26.6%)	6 (14.6%)	0.11
Pulmonary fibrosis	61 (43.9%)	18 (43.9%)	0.10
Pulmonary arterial hypertension	7 (5.0%)	4 (9.8%)	0.27
Renal crisis	0 (0.0%)	0 (0.0%)	–
**Investigation at onset**			
mRSS (points); mean ± SD	7.76 ± 6.44	14.83 ± 10.93	< 0.001*
Anemia	52 (37.7%)	29 (70.7%)	< 0.001*
Serum albumin (g/dL) ; mean ± SD	4.10 ± 0.5	3.84 ± 0.4	0.003**
FVC (% predicted); mean ± SD	71.62 ± 14.40	67.45 ± 13.27	0.14
LVEF < 50%	1 (0.9%)	1 (2.9%)	0.38
**Body composition**			
Lean mass (kg)	37.73 ± 7.64	31.85 ± 5.21	< 0.001*
Fat free mass index (kg/m^2^)	15.79 ± 2.14	13.55 ± 1.39	< 0.001*
Low fat free mass index	7 (5.0%)	29 (70.7%)	< 0.001*
**Medication at onset**			
Prednisolone			
None	56 (40.3%)	10 (24.4%)	–
≤ 15 mg/day	78 (56.1%)	29 (70.7%)	0.01**
> 15 mg/day	5 (3.6%)	2 (4.9%)	0.4
**Immunosuppressive drugs at onset**			
Oral cyclophosphamide	39 (28.1%)	13 (31.7%)	0.53
Methotrexate	10 (7.2%)	5 (12.2%)	0.27
Azathioprine	1 (0.7%)	0 (0.0%)	NA
Mycophenolate mofetil	4 (2.9%)	1 (2.4%)	0.98
**Clinical at enrollment**			
Low body mass index	16 (11.5%)	29 (70.7%)	< 0.001*
Raynaud’s phenomenon	71 (51.1%)	28 (68.3%)	0.05
Digital ulcer	15 (10.8%)	4 (9.8%)	0.85
Telangiectasia	17 (12.2%)	9 (21.9%)	0.12
Calcinosis cutis	2 (1.4%)	2 (4.9%)	0.19
Salt and peppers skin	35 (25.2%)	14 (34.2%)	0.26
Edematous skin	11 (7.9%)	4 (9.8%)	0.71
Tendon friction rub	4 (2.9%)	2 (4.9%)	0.53
Hand deformity	16 (11.5%)	11 (26.8%)	0.02**
Synovitis	2 (1.4%)	1 (2.4%)	0.66
Esophageal involvement	33 (23.7%)	11 (26.8%)	0.69
Stomach involvement	30 (21.6%)	8 (19.5%)	0.78
Pulmonary fibrosis	68 (48.9%)	22 (53.7%)	0.59
Pulmonary arterial hypertension	16 (11.5%)	4 (9.8%)	0.75
Renal crisis	0 (0.0%)	1 (2.4%)	0.06
**Investigation at enrollment**			
mRSS (points); median (IQR)	2 (0–4)	2 (0–9)	0.04**
Anemia	55 (39.6%)	27 (65.9%)	< 0.001*
FVC (% predicted); mean ± SD	69.63 ± 15.03	65.85 ± 14.89	0.20
LVEF < 50%	2 (2.9%)	1 (3.9%)	0.83
High ESR	88 (63.3%)	30 (73.7%)	0.24
High CRP	34 (24.8%)	20 (48.8%)	< 0.001*
Serum albumin (g/dL)	4.4 (4.1–4.6)	4.1 (3.9–4.4)	0.001*
Testosterone level (mIU/L) mean ± SD	5.23 ± 1.93	4.72 ± 1.13	0.28
Estradiol level (ug/dl); mean ± SD	18.36 ± 33.07	16.11 ± 30.51	0.90
Vitamin D insufficiency	68 (48.9%)	10 (24.4%)	< 0.001*
Low Cortisol	27 (19.4%)	13 (31.7%)	0.10
High TSH	12 (8.7%)	9 (21.9%)	0.02**
**Medication at onset**			
Prednisolone			
None	77 (55.4%)	17 (41.5%)	–
≤ 15 mg/day	62 (44.6%)	24 (58.5%)	0.12
> 15 mg/day	0 (0.0%)	0 (0.0%)	NA
Oral cyclophosphamide	16 (11.5%)	6 (14.6%)	0.51
Methotrexate	7 (5.0%)	1 (2.4%)	0.58
Azathioprine	2 (1.4%)	0 (0.0%)	NA
Mycophenolate mofetil	23 (16.6%)	10 (24.4%)	0.26

*95%CI* 95% confidence interval, *NA* data not available due to statistical limitation, *dcSSc* diffuse cutaneous systemic sclerosis, *ATA* anti-topoisomerase I antibody, *ACA* anti-centromere antibody, *mRSS* modified Rodnan skin score, *FVC* forced vital capacity, *LVEF* left ventricular ejection fraction, *IQR* interquartile range, *ESR* erythrocyte sedimentation rate, *CRP* C-reactive protein, *TSH* thyroid stimulating hormone.

*p < 0.001, **p < 0.05.

After doing the univariate analysis, the covariates significantly associated with sarcopenia in SSc patients were included in the multivariate model (Table 3). After adjusting for the significant variables from the univariate logistic regression analysis and relevant clinical confounders, high CRP at enrollment (OR 3.18, 95% CI 1.06–9.54) and low BMI at onset (OR 0.60, 95% CI 0.48–0.75) remained significantly associated with sarcopenia in SSc patients.

**Table 3:** Multivariate logistic regression analysis.

Variable	Crude OR (95% CI)	Adjusted OR (95% CI)
Age at enrollment	1.03 (0.41–2.63)	0.99 (0.94–1.05)
Male sex	2.59 (1.19–5.64)	2.19 (0.77–6.27)
dcSSc subset	3.19 (1.41–7.61)	2.31 (0.67–7.97)
Duration of disease every 1 year	0.99 (0.89–1.11)	1.02 (0.91–1.15)
BMI at onset	0.59 (0.48–0.74)	0.60 (0.48–0.75)*
Anti-topoisomerase I antibody	4.83 (1.11–43.65)	3.16 (0.39–25.92)
High CRP	2.89 (1.30–6.33)	3.18 (1.06–9.54)*
Vitamin D insufficiency	0.34 (0.14–0.78)	0.47 (0.14–1.59)
Anemia at onset	3.99 (1.78–9.32)	2.39 (0.87–6.54)
Prednisolone at enrollment	1.75 (0.82–3.80)	1.04 (0.91–1.18)

*95%CI* 95% confidence interval, *dcSSc* diffuse cutaneous systemic sclerosis, *BMI* body mass index, *ESR* erythrocyte sedimentation rate, *CRP* C-reactive protein.

*p < 0.001.

## Discussion

According to the AWGS definition, we found that the prevalence of sarcopenia was 22.8% (95% CI 1.21–3.48) among Thai SSc patients. There was no significant difference with respect to age and disease duration between patients with and without sarcopenia. The prevalence of sarcopenia in the study was comparable to a previous study conducted in Germany (22.5%)^4^. Our study likewise did not find any differences in age and disease duration between patients with and without sarcopenia. The previous study, however, included 17 cases of mixed connective tissue disease or undifferentiated connective tissue disease, and the majority had lcSSc (54.3%) while the majority in our study had dcSSc; thus, the study populations were different. The SSc subset does not influence the comparison of the prevalence of sarcopenia between the Thai and German SSc patients. The comparable prevalence of sarcopenia is supported by our logistic regression analysis that indicated that neither the dcSSc nor lcSSc subset was associated with sarcopenia.

In general, then, sarcopenia is an age-related disease. The prevalence of sarcopenia in healthy adults age ≥ 60 years—using the definitions proposed by the EWGSOP and AWGS—are around 10% (95% CI 8–12%) in men and 10% (95% CI 8–13%) in women^4^. According to our findings, the prevalence of sarcopenia in SSc appeared to be higher than in the healthy population for people over the age of 60. Furthermore, nearly half of our SSc patients in the younger age group had sarcopenia. There was no age-matched control group in the study. As a result, it is impossible to conclude whether the prevalence of sarcopenia was higher in SSc than in control. However, we suggest that attending physicians should be aware of the risk of sarcopenia in SSc patients, not just the elderly, but also the middle-aged.

Unexpectedly, we found that the severity of disease in SSc patients was not associated with sarcopenia despite their being a mortality risk (viz., extensive skin tightness evaluated by mRSS, internal organ fibrosis (pulmonary fibrosis and gastrointestinal involvement), and vasculopathy (renal crisis and pulmonary arterial hypertension)^26^. The factor that might be a complication or related to SSc disease itself—i.e., low BMI at onset—was a risk for sarcopenia development in SSc. Notwithstanding, the relationship between sarcopenia and SSc is complex and remains uncertain. The pathophysiological process of sarcopenia in SSc is thought to be skeletal muscle involvement, especially muscle weakness, which was revealed in up to 90% of SSc patients^27,28^. Other possible mechanisms include microangiopathy, inflammatory infiltration, and muscular interstitial fibrosis^28^. Malnutrition status from gastrointestinal involvement, chronic inflammation, physical inactivity, medical treatment, and organ failure might also contribute to the development of sarcopenia in SSc^5^. Our study revealed that low BMI at onset and high CRP at enrollment were associated with sarcopenia. This finding supports the role of malnutrition and inflammation in the development of sarcopenia in SSc patients. According to our study design (a cross-sectional study), we can only speculate upon the progression and/or outcome of sarcopenia in SSc patients in cases of improved nutritional status. A longitudinal study on the outcome of sarcopenia among SSc patients would provide important information.

The literature includes some reports of atherosclerotic-related diseases in sarcopenia, particularly among Japanese and Korean patients^29,30^. Hypertension, diabetes mellitus, cardiovascular disease, and chronic kidney disease were reported to be associated with sarcopenia^29–31^. Another study revealed that sarcopenia was associated with multiple comorbidities, cigarette smoking, and low serum levels of testosterone in men^11^. According to the present study, we did not find any significant associations between sarcopenia and comorbidity, cigarette smoking, testosterone level, or medical treatment in SSc patients (particularly steroids and/or immunosuppressants).

The present findings should be interpreted in light of some potential limitations. First, there was no definition for sarcopenia in SSc patients, thus we adapted the definition from the AWGS. The resulting prevalence might not be repeated using other definitions and/or methodologies. Second, there lacked a control group. Therefore, it is impossible to conclude whether the prevalence of sarcopenia was higher in SSc than in control. Third, we did not include patients with active myositis which would be an overlap syndrome (SSc with polymyositis); hence, we cannot examine the correlation of recent or current myositis and sarcopenia. Forth, we did not confirm adrenal insufficiency by adrenocorticotropic hormone (ACTH) stimulation test, so we cannot state the degree of adrenal insufficiency in our patients or whether there is any difference in adrenal insufficiency between patients with and without sarcopenia. Fifth, due to facility limitations, we do not have data on malabsorption for all SSc patients. Finally, we did not examine the correlation between disease activity and sarcopenia, and further study is needed.

The strengths of the study follow. The study enrolled the correct number of patients according to the sample size calculation for answering both the study’s primary and secondary objectives, thus validating the co-prevalence of having SSc and sarcopenia. We also included parameters of interest into the analysis for possible factors associated with sarcopenia in SSc, namely: SSc subset, clinical characteristics of SSc, serology, inflammatory markers, sex hormone, thyroid hormone, adrenal hormone, coexisting diseases and concomitant medications. Our findings have value for evaluating sarcopenia development in SSc patients and could provide insights into giving patients better care and planning future studies.

## Conclusion

Sarcopenia is common in SSc patients and has impact on muscle strength, as well as muscle and physical function. Abnormal body composition is also frequently found in patients with sarcopenia. SSc patients with a low BMI and a high CRP are at risk of sarcopenia and should be monitored closely. Early detection of sarcopenia could lead to preventive measures with the aim of avoiding disability, impaired functional status, and hospitalization.

## Data Availability

The datasets used and/or analyzed during the current study are available from the corresponding author upon reasonable request.
